# Supplementary material for: Comparative genomics provides new insights into the diversity, physiology, and sexuality of the only industrially exploited tremellomycete: Phaffia rhodozyma
Source: BMC Genomics. 2016 Nov 9;17:901. doi: 10.1186/s12864-016-3244-7 (PMC5103461; doi:10.1186/s12864-016-3244-7)
Supplement: Additional file 6: — List of orphan genes with links to PFAM (related to Additional file 1: Table S1). (ZIP 1428 kb) [file 12864_2016_3244_MOESM6_ESM.zip › PFAM_UNIQUE/G04263_P.html]

#                                                                            --- full sequence --- -------------- this domain -------------   hmm coord   ali coord   env coord
  
# target name        accession   tlen query name           accession   qlen   E-value  score  bias   #  of  c-Evalue  i-Evalue  score  bias  from    to  from    to  from    to  acc description of target
  
#------------------- ---------- ----- -------------------- ---------- ----- --------- ------ ----- --- --- --------- --------- ------ ----- ----- ----- ----- ----- ----- ----- ---- ---------------------
  
G04263\_P             -            453 FAD\_binding\_2        PF00890.19   417       9.8    3.5   8.1   1   1     0.084        19    2.6   8.1    36   151   321   434   314   447 0.66 - FAD binding domain  
G04263\_P             -            453 Gly-zipper\_Omp       PF13488.1     46    0.0045   15.3   2.7   1   4       4.6   4.6e+03   -4.0   0.2     9    14   136   141   130   147 0.50 - Glycine zipper  
G04263\_P             -            453 Gly-zipper\_Omp       PF13488.1     46    0.0045   15.3   2.7   2   4   4.5e-06    0.0045   15.3   2.7    16    40   230   254   226   260 0.84 - Glycine zipper  
G04263\_P             -            453 Gly-zipper\_Omp       PF13488.1     46    0.0045   15.3   2.7   3   4         6     6e+03   -6.4  16.2    10    36   308   335   306   347 0.42 - Glycine zipper  
G04263\_P             -            453 Gly-zipper\_Omp       PF13488.1     46    0.0045   15.3   2.7   4   4         1     1e+03   -1.9   0.1    16    27   361   372   359   375 0.60 - Glycine zipper
